# The antihypertensive effect of ethyl acetate extract from red raspberry fruit in hypertensive rats

**DOI:** 10.4103/0973-1296.75885

**Published:** 2011

**Authors:** Han Jia, Ji Wen Liu, Halmurat Ufur, Geng Sheng He, Hai Liqian, Peipei Chen

**Affiliations:** *Department of Nutrition and Food Hygiene, College of Public Health, Xinjiang Medical University, Urumqi, China*; 1 *Department of Pharmacology, College of Pharmacy, Xinjiang Medical University, Urumqi, China*; 2 *Department of Nutrition and Food Hygiene, College of Public Health, Fu Dan University, Shanghai - 200 032, China*; 3 Department of Pharmaceutical Analysis, College of Pharmacy, Xinjiang Medical University, Urumqi, China

**Keywords:** Antioxidants, nitric oxide, raspberry, spontaneously hypertensive rats

## Abstract

**Objectives:**

To evaluate the antihypertensive effect of Xinjiang red raspberry fruit ethyl acetate extract (EER) on spontaneously hypertensive rats (SHR) and its possible mechanism from antioxidant perspective.

**Materials and Methods:**

The SHR rats were randomly divided into 3 groups, and treated with EER low dose (EERL, 100 mg/kg/d), high dose (EERH, 200 mg/kg/d), and water (SHR) through gastric gavage daily for 5 weeks. Another 8 age-matched male Wistar–Kyoto rats were used as normotensive group (WKY). The systolic blood pressure (SBP) was measured by noninvasive tail-cuff method once a week. At the end of the treatment, blood samples were collected and serum concentrations of nitric oxide (NO), superoxide dismutase (SOD), malondialchehyche (MDA), and plasma endothelin (ET) were determined.

**Results:**

Treatment of SHR rats with EER lowered the blood pressure compared with that treated with water (SHR), and the high dose showed more significant reduction in blood pressure. Treatment of SHR rats with EER increased serum NO and SOD levels and lowered ET and MDA levels. As compared with control group, NO levels were increased significantly in EERL (*P* < 0.01), SOD was elevated more significantly in both EERL and EERH (*P* < 0.01); MDA was decreased significantly in EERH group (*P* < 0.05), whereas plasma ET decreased more significantly in the EERH group (*P* < 0.05).

**Conclusions:**

The red raspberry extracts demonstrated a dose-dependent antihypertensive effects in SHR and this may be related to increased NO activation and improved vascular endothelial dysfunction via antioxidation. These results confirmed that raspberries rich in polyphenols have potential cardiovascular protective effects.

## INTRODUCTION

Hypertension is a major public health problem worldwide, when untreated, predisposes to cardiovascular morbidity and premature death.[1,2] As antihypertensive drugs have some side effects, many studies have been conducted to find more suitable antihypertensives from natural sources, such as herbal medicine or components derived from food. Numerous epidemiologic studies have indicated that high intake of fruits and vegetables reduce the risk of cardiovascular diseases.[3] The beneficial effect of fruits and vegetables on the cardiovascular system may be partly explained by the antioxidant effect of the polyphenolic compounds through the pathways that lead to the generation of nitric oxide (NO) by vascular endothelium.[4,5]

The endothelium plays a pivotal role in the regulation of vascular tone and blood pressure by regulating the release of relaxing factors, such as NO, and vasoconstrictor factors, such as endothelin (ET), under basal conditions and after stimulation with vasoactive substances or physical stimuli.[6] Many animal and human studies suggest that an imbalance between NO and ET (ET-1) may contribute to changes in vascular tone observed in hypertension, diabetes, and atherosclerosis.[7,8] Studies also found that excess vascular and extravascular production of reactive oxygen species (ROS), such as superoxide (O_2_^•−^) impairs endothelial NO functions through directly damaging the endothelial cells and by chemical quenching of NO as well.[9,10] Consequently, the loss of NO bioactivity associated with increased vascular superoxide plays a potentially important role in the pathogenesis of endothelial dysfunction. So the factors that can enhance or protect the endothelial NO system, or scavenge and inactivate ROS, have the potential for far-reaching beneficial impacts on cardiovascular diseases.

Polyphenols are the most abundant antioxidants in the diet and richly exist in various berries.[11] Raspberry (*Rubus idaeus*) is one of the important berries rich in polyphenols. In Xinjiang region, the Tianshan and the Altai Mountains are rich in raspberries. Local Mongolian herdsmen in this region use these berries both as food and medicine (especially roots for hypertension and hepatitis) on a daily basis. *In vitro* experimental studies showed that red raspberry fruits have strong activities of scavenging free radicals, inhibiting lipid oxidation, and endothelium-dependent vasodilation,[12–14] indicating that raspberry may have potential protective effect on cardiovascular diseases. Our previous study indicated that crude extracts of red raspberry fruits from Xinjiang region have high content of phenols, and ethyl acetate extracts of raspberry (EER) exhibited the highest antioxidant activity among different polar solvent extracts.[15] Based on the previous studies, we hypothesized that the EER have an antihypertensive effect. To test this hypothesis, we treated spontaneously hypertensive rats (SHR) with EER at two different doses via oral administration daily for 5 weeks and explored its possible mechanisms from antioxidant perspective. Our results demonstrated that EER has antihypertensive effects possibly by inducing increased NO production by vascular endothelial cells and cardiovascular protective effects.

## MATERIALS AND METHODS

### Plant material

The fruits of red raspberry were collected from the local mountains in YiLi district of Xinjiang Province in China. Its identity was confirmed by Dr. Parda from the College of Pharmacy, Xinjiang Medical University. Red raspberry fruit was extracted with 95% ethanol, and further partitioned with petroleum ether, ethyl acetate, and butanol successively. Then EER was used in the present study and its dose was defined as mass (mg) of EER per kg body weight of the animal.

### Animals

Twenty-four 15-week-old male SHR and 8 age-matched male WKY rats were obtained from Shanghai Laboratory Animal Center (SLAC). The rats were maintained at 5 per cage at a constant temperature (22 ± 1°C), with a 12-h dark/light cycle and on standard rat chow. After 1 week of acclimatization to the environment, the SHR rats were randomly divided into 3 groups: control group (SHR), EER low dose group (EERL, 100 mg/kg/d) and high dose group (EERH, 200 mg/kg/d). Another 8 WKY rats were used as normotensive control group (WKY). All the rats were treated daily by gastric gavage with 20 mL/kg body weight of either tap water (SHR and WKY), or an equal volume of distilled water containing respective doses of EER (EERL and EERH) for 5 weeks. During the experimental period, all rats had free access to tap water and chow. Body weight was measured daily.

### Blood pressure measurement

Systolic Blood Pressure (SBP) was measured by indirect tail plethysmography using a computerized instrument (SMUP-PC) from Jialong, Shanghai. This provided an indirect measure of SBP in conscious and slightly warmed rats.[16] Briefly, after prewarming the animals at 38°C for 15 min, SBP were measured in the morning (between 8 and 10 am) 3 times before the initiation of the experiments and during the experiments, SBP was measured once a week and at least 7 determinations were made in every session and the mean of the lowest 3 values within 5 mmHg was taken as the SBP level.[17]

At the end of the treatment, the animals were anesthetized with 4% pentobarbital sodium (1 mL/kg body weight) and the blood samples were obtained from the abdominal aorta into Vaccutainer tubes with EDTA or into regular tubes. All the tubes were centrifuged at 4,000 rpm at 4°C for 10 min and stored at −20°C until determination of serum NO, superoxide dismutase (SOD), malondialchehyche (MDA), and plasma ET levels. NO was detected by nitrate reductase test, MDA by thiobarbituric acid test, and SOD by xanthine oxidase test using commercially available kits (Nanjing Jiancheng, China). ET levels were measured with an ELISA immunoassay kit (ALD goat antirat Endothelin Elisa, Groundwork Biotechnology Diagnosticate Ltd. USA). All the tests were performed according to the manufacturer’s instructions.

### Data analysis and statistics

Results are shown as mean ± SEM from 8 rats. Changes in the blood pressure were compared using Student’s *t* test. The differences between the groups were compared using one-way ANOVA test, and a value of *P* < 0.05 was considered statistically significant.

## RESULTS

### Antihypertensive effects of red raspberry fruit extracts

**Figure 1:**
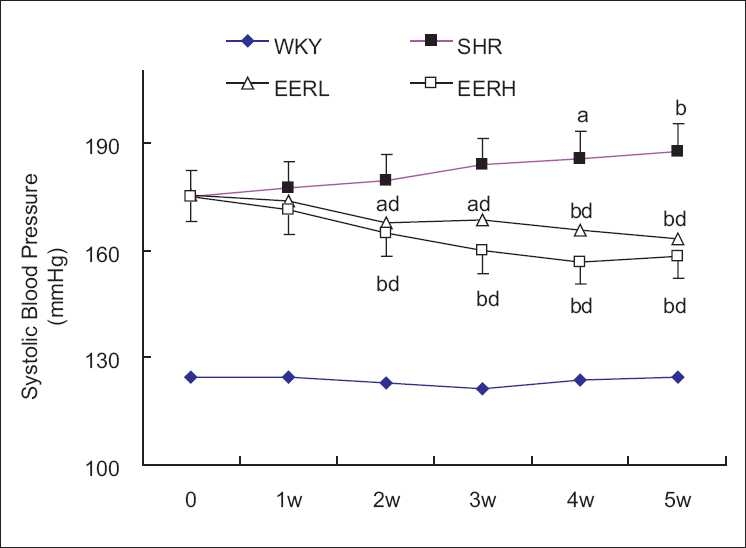
Changes in SBP of experimental rats during the 5-week treatment period. Rats receiving intragastric administration of water (WKY, SHR) and EER (EERL, 100 mg/kg/d, EERH, 200 mg/kg/d) daily for 5 weeks. SBP in the SHR (■), EERL (∆), EERH (□), and WKY(♦) groups. Data are means ± SEM. Compared with level, ^a^*P* < 0.05 vs baseline, ^b^*P* < 0.01 vs baseline, ^c^*P* < 0.05 vs SHR, ^d^*P* < 0.01 vs SHR. SBP, systolic blood pressure; SHR, spontaneously hypertensive rats; WKY, Wistar–Kyoto rats; EERL, EER low dose; EERH, EER high dose

The results from this study, as illustrated in Figure 1 and summarized in Table 1, demonstrated that at the start of the experiment, the mean values of SBP in all the SHR were similar (174.43 ± 5.27 to 175.09 ± 6.72 mmHg). The treatment of SHR with EER started to reduce SBP after treatment for 1 week, and this effect continued until the 5-week treatment period with gradual reduction in SBP. Among the two doses of treatment, EERH reduced the SBP more significantly at week 4 and 5 compared with the SHR (treated with water only). During the 5-week treatment period, the SBP in SHR was slowly increased up to 187 mmHg at the end of the experiment, but there were no obvious changes in the SBP in WKY. These experiments suggested that EER treatment reduced the SBP in a time- and dose-dependent manner with specificity against hypertensive rats.

**Table 1:** Changes in SBP during the treatment (*x* ± *s* mmHg)

Group	n	Before intervention	After intervention				
			Week 1	Week 2	Week 3	Week 4	Week 5
WKY	8	124.37 ± 4.84	124.34 ± 6.16	122.85 ± 4.80	121.38 ± 4.83	123.53 ± 5.83	124.62 ± 4.78
SHR	8	175.09 ± 6.72	177.47 ± 6.34	179.49 ± 7.59	183.81 ± 9.32	185.67 ± 8.07^a^	187.67 ± 7.45^b^
EERL	8	175.21 ± 4.24	173.14 ± 6.65	167.70 ± 7.02^a^^d^	168.25 ± 5.09^a^^d^	165.25 ± 7.33^b^^d^	162.98 ± 5.49^b^^d^
EERH	8	174.43 ± 5.27	171.76 ± 4.94	163.38 ± 5.46^b^^d^	159.81 ± 5.57^b^^d^	156.66 ± 4.15^b^^d^	158.37 ± 6.90^b^^d^

SHR: Spontaneously hypertensive rats, WKY: Wistar–Kyoto rats, EERL: EER low dose, EERH: EER high dose

a *P* < 0.05 vs baseline

b *P* < 0.01 vs baseline

c *P* < 0.05 vs SHR

d *P* < 0.01 vs SHR

### Effect of EER on serum NO and plasma ET levels

To understand the underlying mechanisms of antihypertensive effect of raspberry EER, we measured serum contents of NO and ET levels. As summarized in Table 2, the basal serum NO levels in the SHR were lower than those of WKY, although the difference is not significant (*P* > 0.05). Plasma ET levels in SHR rats were higher than those of WKY (*P* < 0.05). Treatment of SHR with EER increased the serum NO levels with both doses, but compared with SHR, EERL increased more significantly (*P* < 0.01). Both doses of EER decreased plasma ET levels, and EERH reduced significantly (*P* < 0.05) as compared with SHR.

**Table 2:** Effect of EER on serum NO and ET in SHR and WKY rats (*x* ± *s*)

Group	n	NO (μmol/L)	ET (ng/mL)
WKY	8	17.60 ± 2.85	1.09 ± 0.21
SHR	8	13.32 ± 5.80	1.35 ± 0.24^a^
EERL	8	24.92 ± 8.74^a^^d^	1.30 ± 0.21
EERH	8	19.13 ± 4.73	1.02 ± 0.17^c^

NO: Nitric oxide, ET: Endothelin, SHR: Spontaneously hypertensive rats, WKY: Wistar–Kyoto rats, EERL: EER low dose, EERH: EER high dose

a *P* < 0.05 vs WKY

b *P* < 0.01 vs WKY

c *P* < 0.05 vs SHR

d *P* < 0.01 vs SHR

### Effect of EER on serum SOD and MDA in SHR and WKY rats

We also examined the serum SOD and MDA content of the rats after treatment and the results are summarized in Table 3. The basal serum SOD level in SHR was lower than that of WKY (*P* < 0.05), but the MDA level in SHR was lower than that of WKY. As compared with SHR, EER significantly increased serum SOD activity at both doses (*P* < 0.01), and decreased MDA levels with significant decrease in the EERH group (*P* < 0.05).

**Table 3:** Effect of EER on serum SOD and MDA levels in SHR and WKY rats (*x* ± *s*)

Group	n	SOD (U/mL)	MDA (nmol/mL)
WKY	8	289.94 ± 5.86^d^	8.68 ± 0.73
SHR	8	273.02 ± 13.29^b^	6.84 ± 0.78^b^
EERL	8	323.20 ± 14.79^b^^d^	6.09 ± 0.63^b^
EERH	8	316.92 ± 11.63^b^^d^	5.95 ± 0.84^b^^c^

SOD, superoxide dismutase; MDA, malondialchehyche; SHR, spontaneously hypertensive rats; WKY, Wistar–Kyoto rats; EERL, EER low dose; EERH, EER high dose

a *P* < 0.05 vs WKY

b *P* < 0.01 vs WKY

c *P* < 0.05 vs SHR

d *P* < 0.01 vs SHR

## DISCUSSION

Red raspberry has been consumed on a daily basis by nomads during summer in rural area of Xinjiang region, and has been even used as remedies for common ailments, including hypertension. Our studies with extracts of raspberry in SHR demonstrated for the first time, to the best of our knowledge, that feeding rats with extracts of raspberry indeed showed SBP reducing effect, especially in the hypertensive rats in dose- and time-dependent manner. The extracts of raspberry increased NO production, enhanced SOD activity, and reduced ET in the serum of the treated rats, suggesting the possible mechanisms for SBP reducing effect of EER via tipping the balance between NO/ET system toward increased vasodilation and improved endothelial physiology.

Although the data generated by animal models could not recapitulate the situation in humans naturally consuming berries, we believe that our animal model applied in this study is a reasonably suitable *in vivo* system for testing the antihypertensive effect of raspberry extracts. Previous reports have demonstrated that endothelium-dependent vasodilatation is impaired in SHR compared with normotensive WKY animals and increased oxidative inactivation of NO by an excess of O_2_^•−^ may account for the decrease in NO availability and endothelial dysfunction seen in SHR.[18–20] Thus, it was reasoned that the SHR was an appropriate model to study the effect of raspberry EER on hypertension.

Hypertension, as many other vascular diseases, associated with an impaired bioavailability of NO, and this has been found to be linked to enhanced synthesis of ET, implying that the imbalance between NO and ET may contribute to changes in vascular tone.[21–23] Consistent with these previous studies, our study found that EER reduced the ET and increased NO in the serum of experimental animals, and this further suggested the possible mechanisms for SBP reducing effects of red raspberry extracts, which may be associated with maintenance of NO/ET balance.

Besides, elevated oxidative stress-mediated destruction of NO also contributes to hypertension. Previous studies also reported that the higher oxidative stress in hypertension was due to imbalance between oxidants and antioxidants as a result of increased oxidant production and decreased antioxidant enzymes.[24,25] An increase of lipid peroxidation products in plasma and increase in ROS generation in the vascular cells was observed in the SHR.[25] Kumar reported an increase in the lipid peroxides with a decrease in the antioxidants, such as catalase and SOD.[26] In this study, SOD activities were lower in SHR compared with WKY rats, but MDA, markers of lipid peroxidation induced by ROS, were lowered in SHR, not consistent with some other report.[17] In a previous study, we found that serum lipid level in WKY was higher than that of SHR, consistent with Yuan’s reports.[27] This may provide clues to partially explain the high level of MDA in WKY compared with that of SHR. Red raspberry EER increased SOD and reduced MDA in serum of SHR, thus suggesting another possible SBP reducing mechanism of EER, and that may partly explains the antihypertensive effect of red raspberry extracts.

Previous *in vitro* and *in vivo* studies suggested that red wines, other grape products, and various berries that contain polyphenols can induce endothelium-dependent vasorelaxation, probably via NO release or by enhancing the biological activity of NO.[28–31] The favorable changes in platelet function, blood pressure, and HDL cholesterol were also found after the consumption of berries for 2 months.[32] The extracts of the leaves and fruits of *Rubus* species have been used in various countries as natural remedies to treat several diseases, such as hypertension and diabetes.[33] In these berries, ellagitannins are the main class of phenolic compounds.[34,35] Potential cardiovascular protective effects of ellagitannins have also been reported, and in vasorelaxation assays on rabbit aorta found that sanguin H-6 and lambertianin C were the active components of raspberry extracts responsible for vasorelaxation activity.[14]

Our previous study showed that raspberry fruits from Xinjiang region have high content of polyphenols, including tannins, flavonoids, and phenolic acids, and have high antioxidant activity. Therefore, the antihypertensive effect of EER could be attributed to the increased levels of NO by an antioxidant effect. But we also found that EER increased serum NO levels with both doses but the low dose increased NO significantly. Previous studies suggested that high quantity of polyphenols could exhibit pro-oxidant properties instead of antioxidant properties.[36] Therefore, we should be concerned about the dose in the future studies. Based on all the above-mentioned previous studies and the findings from the current study, it is both tempting and reasonable to speculate that increased uptake of raspberries by patients with hypertension as an additive to the standard prescribed antihypertension therapy would result in improved treatment outcome.

In conclusion, we found that the treatment of SHR with raspberry fruit EER induced a progressive decrease in the blood pressure. Since EER substantially increased SHR serum NO level and decreased plasma ET level, we believe that the possible mechanism of antihypertensive effect of EER on SHR may be related to the endothelium-dependent vasorelaxation. Besides, EER increased serum SOD activity, decreased MDA level, suggesting that reducing oxidative stress in the circulation may also play a role as part of the mechanisms. Our results suggest a possible beneficial effect of raspberry on cardiovascular function and thus provided mechanistic basis for its use as antihypertensive remedies in folk medicine.

However, precise description of antihypertensive mechanism of the EER needs further studies aimed at evaluating the effect of EER on the endothelial NADH/NADPH-dependent enzymatic sources of superoxide production system, and identifying active components of EER with antihypertensive effect.
